# Coherent control of optical spin-orbit interactions

**DOI:** 10.1126/sciadv.aec4604

**Published:** 2026-04-10

**Authors:** Hongwei Yang, Kai Tu, Zhuguang Chen, Yao Hong, Jieyuan Tang, Huadan Zheng, Yongchun Zhong, Jianhui Yu, Zhe Chen, Wenguo Zhu

**Affiliations:** ^1^ Key Laboratory of Optoelectronic Information and Sensing Technologies of Guangdong Higher Education Institutes, Department of Optoelectronic Engineering, Jinan University, Guangzhou 510632, China.; ^2^ JiHua Laboratory, Foshan, Guangdong 528200, China.

## Abstract

Optical spin-orbit interactions (SOI) are an intriguing physical phenomenon where the spin (circular polarization) of light influences and governs its spatial degrees of freedom. Yet, achieving real-time and flexible control over SOI remains a formidable challenge. Here, we propose a coherent control method for the flexible manipulation of optical SOI using a thin isotropic crystal. The output horizontally and vertically polarized waves from the crystal exhibit strong and distinct dependencies on the phase delay between the input signal and control waves, leading to substantial spin-orbit beam shifts. By tuning the relative phase between the signal beam and the control beam, we observe transitions from Gaussian to first-order orbital angular momentum (OAM) vortex modes. The achievable transition speed reaches 11.5 gigahertz, now limited by the external electro-optic link in the control arm. Notably, the dual-port architecture offers coherent and reversible selection of spin-OAM states, with the output port uniquely determined by the phase delay between two counter-propagating input beams. This ultrafast coherent control method opens avenues for advanced manipulation of SOI.

## Teaser

Ultrafast coherent control enables flexible manipulation of optical spin-orbit interactions in a thin isotropic crystal.

## INTRODUCTION

Spin-orbit interaction (SOI) of light arises from the fundamental vectorial nature of electromagnetic waves, embodying the intrinsic coupling between spin angular momentum, associated with polarization, and orbital angular momentum (OAM), stemming from the spatial structure of the optical field (*1*). This interaction becomes particularly prominent in structured or inhomogeneous media, tightly focused beams, and at interfaces where the transversality condition imposes helicity-dependent geometric phase shifts (*2*–*8*). Analogous to the SOI in relativistic quantum systems, optical SOI governs phenomena such as spin-dependent beam shifts (e.g., Imbert-Fedorov and photonic Spin Hall effects) (*9*–*11*), spin-to-OAM conversion (*12*, *13*), and spin-controlled directional coupling (*14*–*16*). These effects, once negligible in macroscopic optics, now play a central role at subwavelength scales, offering powerful mechanisms for light manipulation in nanophotonics, metasurfaces, and quantum optical systems (*17*–*20*). However, these SOI systems are inherently limited in adaptability, as their spatial configurations and optical responses are fixed during fabrication, resulting in operation confined to static regimes.

Driven by rapid advances in material science and micro/nanofabrication, researchers have increasingly integrated externally responsive materials into SOI architectures, leading to a new class of actively reconfigurable and programmable SOI systems (*21*–*27*). Notable examples include electro-optic crystals (*21*, *22*), ferroelectric nematic liquid crystals (*23*–*26*), and phase-change materials (*27*–*29*), which exhibit reversible tunability in optical properties when subjected to external electric, magnetic, or mechanical stimuli. These materials open up possibilities for dynamic modulation of SOI phenomena.

In addition to device-level modulation strategies, SOI can also be dynamically influenced by tailoring the properties of the incident light itself. By engineering parameters such as polarization state (*30*), or temporal coherence (*31*, *32*), it is possible to reshape the interaction landscape without modifying the photonic structure. These input-based schemes offer a versatile degree of control, enabling tunable SOI responses through adjustments in incident conditions. Such approaches are particularly advantageous in integrated platforms, where reconfigurability and minimal structural complexity are essential.

Despite these advances, most existing approaches to SOI control remain constrained by limited modulation speeds, complex fabrication requirements, or the need for anisotropic and nanostructured materials. A critical challenge persists in realizing fast, real-time, and tunable manipulation of SOI effects within compact and scalable architectures. So far, no demonstrated platform has achieved dynamic SOI control at gigahertz-level modulation speeds (*21*–*30*), which poses a substantial barrier for integration into high-speed photonic systems requiring ultrafast and reconfigurable functionality.

In this context, coherent control offers a compelling solution (*33*–*44*). Rather than tailoring the material properties of the interface, coherent control reconfigures multiport scattering by programming the relative phase, amplitude, or polarization of multiple coherent inputs. This interferometric paradigm is well established, with coherent perfect absorption (CPA) as a canonical example (*37*–*42*). CPA was originally formulated as a time-reversed laser (*37*) and experimentally demonstrated as interferometric control of absorption (*38*), with subsequent extensions such as exceptional-point and wavefront-selective CPA (*39*, *40*) and broader reviews formalizing “linear control of light with light” (*42*). Beyond absorption, coherent control has also been extended to multiport scattering engineering such as coherent perfect diffraction (CPD) in meta-gratings (*43*) and coherent full polarization control enabled by bound states in the continuum (BICs) (*44*). Extending coherent control to the domain of SOI opens opportunities for dynamically and reversibly modulating spin-dependent beam properties.

In this work, we propose and demonstrate a coherent control strategy for actively modulating optical SOI using a single isotropic crystal. The key mechanism relies on the interference between two obliquely incident beams—a signal and a control beam—with adjustable relative phase. By tuning the phase delay, we achieve manipulation of the spatial distribution of spin-resolved fields, leading to pronounced spin-orbit beam shifts and mode transitions governed by SOI. We observe a coherent phase-controlled spin-dependent routing of spin-OAM states in a dual-port configuration, with the output port of a given spin-OAM state determined by the sign of the phase delay. The measured system bandwidth, reaching 11.5 GHz (3 dB), is determined by the combined response of the external electro-optic link, rather than by the material response of the optical interface. These results establish a fast, reconfigurable, and material-agnostic platform for dynamic SOI control, offering a promising route toward spin-dependent beam shaping and programmable structured light generation in integrated photonic systems.

## RESULTS

### Coherent phase-driven SOI modulation

**Fig. 1.**
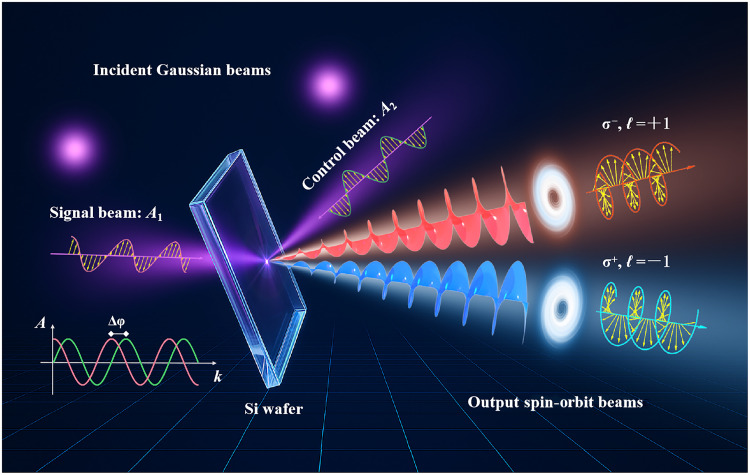
Schematic of coherent SOI control. Two counter-propagating beams (unstructured Gaussian beams: *A*_1_ as signal and *A*_2_ as control) are simultaneously launched onto the wafer, with a tunable relative phase delay Δφ governing their interference dynamics. Spin-dependent first-order OAM beams are generated, resulting from coherent SOI modulation.

As illustrated schematically in Fig. 1, two counter-propagating beams, referred to as the signal beam *A*_1_ and the control beam *A*_2_, are launched simultaneously into the wafer via two ports (indexed by *j* = 1,2) with a tunable relative phase delay Δφ. The corresponding output fields are denoted as *B_j_*. The relation between the input and output is related by the scattering matrix **S** with B=SA (*38*, *42*), where the Jones vectors $A=A_{j}^{p,s}$ and $B=B_{j}^{p,s}$ represent the complex amplitude of the input and output fields for both ports and polarization components. In detail, the scattering matrix **S** is block-diagonal, reflecting the absence of cross-polarization coupling in an isotropic slab

$$S=\left[\begin{array}{cc} S^{P} & 0\\ 0 & S^{s} \end{array}\right],S^{p,s}=\left[\begin{array}{cc} r_{p,s} & t_{p,s}\\ t_{p,s} & r_{p,s} \end{array}\right]$$ (1)

Here, $r_{p,s}$ and $t_{p,s}$ denote the Fresnel reflection and transmission coefficients for horizontally (*p*) and vertically (*s*) polarized incidence, respectively.

For a monochromatic plane wave incident from air (*n*_0_ = 1) onto a Si slab at an angle θ_1_, the optical response is governed by the complex refractive index $n=n^{'}+i\kappa$ at λ = 1064 nm. For compact analytical derivations, we present closed-form expressions in the lossless limit (κ → 0); however, all numerical calculations, simulations, and fittings reported in this work were performed using the full complex refractive index $n=n^{'}+i\kappa$ of Si. The refraction angle θ_2_ inside the wafer follows Snell’s law as $\theta_{2}=\text{asin}\left(\text{sin}\theta_{1}/n\right)$. At the air-wafer interface, the Fresnel reflection and transmission coefficients $r_{p,s}^{\text{intf}}$ and $t_{p,s}^{\text{intf}}$ depend on θ_1_ and are determined by the standard electromagnetic boundary conditions. To account for the finite thickness *d* of the wafer, a single internal traversal inside the wafer introduces a cumulative phase $\delta =2\mathrm{\pi n}\text{cos}\theta_{2}d/\lambda$. Cases involving absorption are discussed in the Supplementary Materials (fig. S1). Multiple internal reflections within the wafer result in Fabry-Pérot–type interference, modifying the effective reflection and transmission coefficients $r_{p,s}$ and $t_{p,s}$ by incorporating both the interface response and the phase accumulation during light propagation inside the wafer (see Materials and Methods for details).

**Fig. 2.**
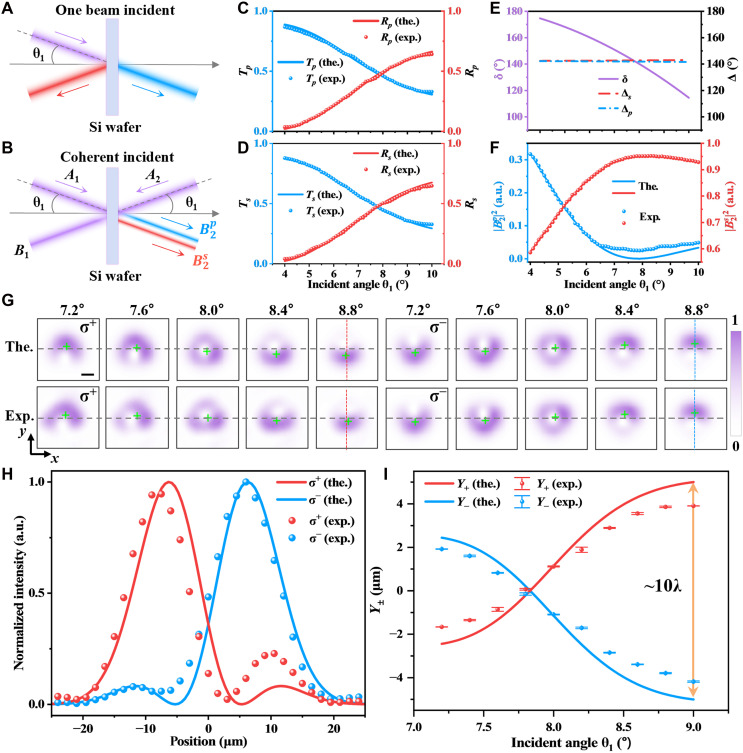
Spin-orbit interaction induced beam evolution and spin-orbit beam shifts under coherent incidence. (**A** and **B**) Schematic of the experimental configuration: (A) single-beam configuration and (B) coherent dual-beam configuration comprising counter-propagating signal *A*_1_ and control *A*_2_ beams. (**C** and **D**) Measured and calculated reflection and transmission spectra (*T*_*p*,*s*_ and *R*_*p*,*s*_) for the single-beam incidence case under horizontally (C) and vertically (D) polarized illumination. (**E** to **I**) Results for the dual-beam configuration with a fixed relative phase delay of Δφ=−π/2. (E) Calculated cumulative phase δ and critical cumulative phase $\Delta_{p,s}$. Intersections mark the incident angle where coherent extinction occurs ($|B_{2}^{p}|=0$) under Δφ=−π/2. (F) Normalized output intensities ${|B_{2}^{p,s}|}^{2}$ under dual-beam coherent interference. (G) Theoretical (top) and experimental (bottom) output intensity patterns for spin states $\sigma^{\mp }$ for different incidence angles θ_1_. Scale bar, 10 μm. (H) Cross-sectional intensity profiles at $\theta_{1}=8.8{}^{\circ}$ for both spin states. (I) Spin-orbit beam shifts $Y_{\pm }$ versus θ_1_. The vertical error bars indicate the standard error (SE) obtained from centroid extractions from independently recorded beam profile images (three measurements). (F to H) Measured and calculated outputs at port 2, and in all dual-beam measurements, both incident beams are horizontally polarized. a.u., arbitrary units; the., theoretical; exp., experimental.

As illustrated in Fig. 2 (A and B), two excitation scenarios are considered. In the single-beam configuration (Fig. 2A), one input wave is launched at an incident angle θ_1_. The resulting reflection and transmission spectra for horizontally and vertically polarized waves are shown in Fig. 2 (C and D), respectively. The spectral profiles exhibit similar overall trends for both polarization states, indicating that their angular response of Si wafer share comparable behavior. Notably, the crossing points between the transmission and reflection curves for both polarizations occur around an incident angle of approximately $\theta_{1}=7.9{}^{\circ}$, suggesting a balanced amplitude of the effective reflection and transmission coefficients at this angle. In contrast, the coherent dual-beam configuration (Fig. 2B) introduces two counter-propagating incident waves $A_{j}$, with a controllable relative phase delay Δφ, enabling coherent interference at the output ports.

In the coherent dual-beam configuration, the total field amplitudes at the front and rear the wafer for each polarization can be expressed as $B_{1,2}^{p,s}=r_{p,s}A_{1,2}^{p,s}+t_{p,s}A_{2,1}^{p,s}$. Assuming two counter-propagating waves of unit amplitude $|A_{j}^{p,s}|=1$ and that two waves have a relative phase of −π/2, constructive or destructive interference can be engineered at the outputs depending on the Fresnel response. Specifically, destructive interference between two counter-propagating waves occurs when the effective Fresnel coefficients are of equal amplitude, leading to complete cancellation of the output field, such that $|B_{1}^{s}|=0$ and $|B_{2}^{p}|=0$. The difference in behavior between two polarization components is caused by the half-wave loss for vertical polarization reflection. These conditions of coherent extinction yield a closed-form expression for the critical cumulative phase $\Delta_{p,s}$, which corresponds to the total phase delay δ accumulated during propagation and satisfies the Fresnel constraint of $\text{sin}\Delta_{p,s}={\left(-1\right)}^{j}\left[1-{\left(r_{p,s}^{\text{intf}}\right)}^{2}\right]/2r_{p,s}^{\text{intf}}$ (see Materials and Methods for details). As shown in Fig. 2E, the cumulative phase δ varies continuously with the incident angle under Δφ=−π/2, whereas the critical cumulative phase $\Delta_{p,s}$ remains nearly constant due to the weak angular dependence of $r_{p,s}^{\text{intf}}$ at small incident angles. Constructive or destructive interference occurs at angular positions where the cumulative phase adheres $\delta =\Delta_{p,s}$. This intersection appears near $\theta_{1}=7.9{}^{\circ}$, which coincides with the angular position at which the effective reflection and transmission amplitudes become equal under single-beam configuration. Figure 2F displays the normalized output intensities at output port 2 for both polarization states as a function of the incident angle. A pronounced dip in the horizontally polarized component ($|B_{2}^{p}|=0$) and a peak in the vertically polarized one are observed near $\theta_{1}=7.9{}^{\circ}$, consistent with the expected destructive and constructive interference, respectively.

Let us consider a Gaussian wave packet with monochromatic frequency impinging from air to the Si wafer. The incident angular spectrum is given by ${\tilde{A}}_{0}=\frac{w_{0}}{\sqrt{2\pi }}\text{exp}\left[-{w_{0}}^{2}\left({k_{x}}^{2}+{k_{y}}^{2}\right)/4\right]$, where $k_{x,y}$ are the transverse wave vectors, as well as *w*_0_ is the radial of the beam waist. The transmitted and reflected angular spectra are related to the incident angular spectrum by means of the relation $\tilde{B}={\hat{T}}_{a}\tilde{A}$ (*9*), where ${\hat{T}}_{a}$ is the transformation matrix (see Materials and Methods for details). By applying the transformation matrix to the two counter-propagating incident beams across the Si wafer, we obtain the complete scattering matrix **S** in the coherent dual-beam configuration, which relates all input and output angular spectral components by

$$S=\left[\begin{array}{cccc} r_{p}+\kappa_{x}r_{p}^{\prime } & t_{p}+\kappa_{x}t_{p}^{\prime } & \kappa_{y}M & \kappa_{y}N\\ t_{p}+\kappa_{x}t_{p}^{\prime } & r_{p}+\kappa_{x}r_{p}^{\prime } & \kappa_{y}N & \kappa_{y}M\\ -\kappa_{y}M & \kappa_{y}N & r_{s}+\kappa_{x}r_{s}^{\prime } & t_{s}+\kappa_{x}t_{s}^{\prime }\\ \kappa_{y}N & -\kappa_{y}M & t_{s}+\kappa_{x}t_{s}^{\prime } & r_{s}+\kappa_{x}r_{s}^{\prime } \end{array}\right]$$ (2)

Here, primes indicate derivatives with respect to the incidence angle, i.e., $r_{p,s}^{\prime }=\partial_{\theta_{1}}r_{p,s}$, $t_{p,s}^{\prime }=\partial_{\theta_{1}}t_{p,s}$. The normalized transverse wave vector components are defined as $\kappa_{x,y}=k_{x,y}/k_{0}$, where $k_{0}$ is the vacuum wave number. The SOI strengths $M=\left(r_{p}+r_{s}\right)\text{cot}\theta_{1}$ and $N=\left(t_{p}-t_{s}\right)\text{cot}\theta_{1}$ control the κ*_y_*-mediated cross-polarization mixing.

Under coherent dual-beam incidence (horizontally polarized), the output field at output port 2 can be described by (see Materials and Methods for detailed derivation) ${\tilde{B}}_{2}^{\left(\sigma \right)}={\tilde{A}}_{\text{eff}}\cdot \left[1-\mathrm{\gamma i}{\sigma \kappa }_{y}+{\beta \kappa }_{x}\right]{\hat{e}}_{\sigma }$, where σ=±1 denotes the spin states, and ${\tilde{A}}_{\text{eff}}={\tilde{A}}_{0}\left(r_{p}e^{i\Delta \varphi }+t_{p}\right)$ is the effective field amplitude. The SOI strength is characterized by the dimensionless complex coupling coefficient $\gamma =\frac{-{\mathrm{Me}}^{i\Delta \varphi }+N}{r_{p}e^{i\Delta \varphi }+t_{p}}$, while the term $\beta =\frac{r_{p}^{\prime }e^{i\Delta \varphi }+t_{p}^{\prime }}{r_{p}e^{i\Delta \varphi }+t_{p}}$ accounts for the angular dispersion of the Fresnel coefficients. In the regime of weak spin-orbit interaction ($|{\gamma \kappa }_{y}|\ll 1$), the output field can be approximated as

$${\tilde{B}}_{2}^{\left(\sigma \right)}\approx {\tilde{A}}_{\text{eff}}\cdot \left[e^{-\mathrm{\gamma i}{\sigma \kappa }_{y}}+{\beta \kappa }_{x}\right]{\hat{e}}_{\sigma }$$ (3)

revealing that spin-orbit beam shifts are induced by the coupling coefficient γ. Hence, the spin-orbit beam shifts can be precisely controlled via the angular response of the Fresnel coefficients and the relative phase. The in-plane phase term β remains spin independent under symmetric excitation yet becomes spin dependent upon breaking the polarization symmetry of the incident dual beams.

Particularly, we consider a specific phase condition of Δφ=−π/2. When the Fresnel coefficients satisfy $t_{p}-{\mathrm{ir}}_{p}=0$, a strong SOI arises. Owing to the π/2 phase difference between the complex coefficients of $\kappa_{x}$ and $\kappa_{y}$, i.e., $\left(-ir_{p}^{\prime }+t_{p}^{\prime }\right)$ and (iM+N), the output field becomes (see Materials and Methods for details)

$${\tilde{B}}_{2}={\tilde{A}}_{0}\left\{|-ir_{p}^{\prime }+t_{p}^{\prime }|\kappa_{x}-\mathrm{i\sigma}|\mathrm{iM}+N|\kappa_{y}\right\}{\hat{e}}_{\sigma }$$ (4)

indicating that opposite topological vortex phases are selectively imparted into the output σ^−^ and σ^+^ spin states, provided that the prefactors of the κ*_x_*- and κ*_y_*-related terms are equal. However, these prefactors are generally unequal, leading to imperfect optical vortexes and the emergent of OAM sidebands.

These SOI-induced effects are directly visualized in Fig. 2 (G to I), where both beams, with a fixed beam waist *w*_0_ of 12 μm, are simultaneously tilted with a tunable incident angle θ_1_. At fixed Δφ = −π/2, the horizontal component of output beam exhibits a distinctive and repeatable pattern versus θ_1_, which is used to be a “fingerprint” for calibrating θ_1_ scans (fig. S2). As shown in Fig. 2G, the output field evolves from vortex-like profiles to beam shifts with opposite transverse displacements for the σ^−^ and σ^+^ spin states. Figure 2H shows, for an incident angle of $\theta_{1}=8.8{}^{\circ}$, the intensity profiles extracted along the blue and red dashed lines in Fig. 2G, clearly resolving the spatial separation between spin components. The theoretical calculated spin-orbit beam shifts $Y_{\pm }$ (see Materials and Methods for derivation) are plotted in Fig. 2I, which shows good agreement with experimental measurements and confirms that the observed spin-orbit beam shift reaches approximately 10λ between the two opposite spin components. Each spin component undergoes a shift of approximately ±5λ (~0.416*w*_0_) with respect to the mean beam position (*45*). The Stokes parameter maps (*46*) across the output beam profiles (figs. S3 and S4) confirm that the observed beam separations are genuinely spin resolved, with a polarization cross-talk lower than −14.5 dB (fig. S5).

**Fig. 3.**
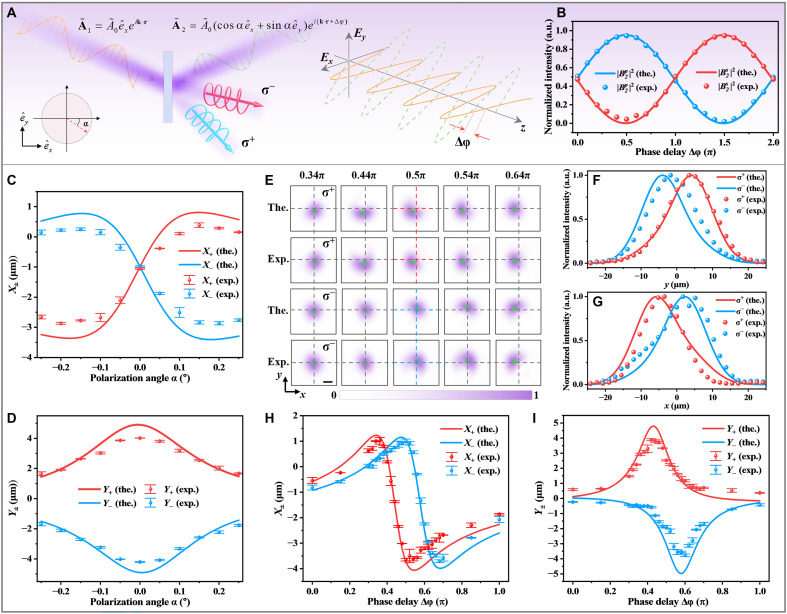
Coherently controlled spin-orbit interaction via polarization and phase tuning. (**A**) Schematic of the experimental configuration. A horizontally polarized signal beam ${\tilde{A}}_{1}$ and a control beam ${\tilde{A}}_{2}$ (linearly polarized at a small angle α from the horizontal) are incident symmetrically on a Si wafer with a tunable relative phase delay Δφ. (**B**) Normalized output intensities at port 2 under varying Δφ for a fixed polarization angle α=0°. (**C** and **D**) Spin-resolved beam centroid shifts along the *x* and *y* axes, respectively, as a function of the polarization angle α for a fixed phase delay Δφ=−π/2. (**E**) Spin-resolved beam profiles under varying Δφ at fixed α=−0.15° and $\theta_{1}=8.8{}^{\circ}$; green crosses indicate centroid positions. Scale bar, 10 μm. (**F** and **G**) Intensity profiles along the *y* and *x* axes, respectively, extracted from the red and blue dashed lines in (E) for Δφ=−π/2, quantifying the beam shifts for each spin component. (**H** and **I**) Spin-resolved beam centroid positions along *x* (H) and *y* (I) versus Δφ. The vertical error bars indicate the SE obtained from centroid extractions from independently recorded beam profile images (three measurements).

Figure 3 illustrates the SOI enabled by coherent control of the polarization and relative phase between the signal and control beams. As schematically illustrated in Fig. 3A, the signal beam **A**_1_ is horizontally polarized, while the polarization state of the control beam is slightly tilted by an angle α, introducing a weak vertical polarization component. Two beams are incident symmetrically onto the Si wafer from opposite directions with a relative phase delay Δφ. At output port 2, this configuration gives rise to pronounced spin-orbit beam shifts, manifested as spatial displacements between the spin states $\sigma^{\mp }$ due to SOI. Figure 3B presents the normalized output normalized intensities ${|B_{p}^{2}|}^{2}$ and ${|B_{s}^{2}|}^{2}$ as a function of Δφ under two counter-propagating plane waves incidence with a fixed polarization angle α=−0.15°. The observed complementary sinusoidal modulation confirms phase-controlled energy redistribution between the two orthogonal polarization components. Specifically, the energy valley is at Δφ=−π/2 for horizontal polarization and appears at Δφ=+π/2 for vertical polarization.

Figure 3 (C and D) shows that the beam shifts along the *x* (in-plane) and *y* (transverse) directions as a function of the polarization angle α under Δφ=−π/2*.* Notably, both the *x*-direction optical shifts and *y*-direction spin-orbit beam shifts exhibit high sensitivity to slight variations in α and reveal opposite trends for the two spin states $\sigma^{\mp }$, indicative of spin-dependent spatial separation. The experimental results well match theoretical predictions, validating the polarization-induced symmetry breaking and the resulting spin-dependent beam shifts.

To further illustrate the coherently controlled SOI-induced spatial evolution, Fig. 3E presents the spin-resolved beam profiles at port 2 under varying phase delay Δφ, with the polarization fixed at α=−0.15° with $\theta_{1}=8.8{}^{\circ}$. Both theoretical and experimental results exhibit clear lateral separation between the σ^−^ and σ^+^ spin states, accompanied by pronounced centroid shifts as Δφ is tuned. The centroid positions of the beam profiles, visually marked by green crosses in Fig. 3E, clearly reveal the spin-dependent evolution of the beam profiles with phase delay. Figure 3 (F and G) presents the intensity profiles along the *y* and *x* axes for Δφ=−π/2, respectively, confirming the directional asymmetry between the two spin states. Figure 3H shows the optical shifts $X_{\pm }$ (see Materials and Methods for details), while Fig. 3I illustrates the spin-orbit beam shifts $Y_{\pm }$, corresponding to the σ^−^ and σ^+^ spin states, respectively. Both beam shifts exhibit strong spin-dependent modulation, with the centroid positions varying nonlinearly with Δφ. Polarization symmetry breaking induces spin-selective shifts $X_{\pm }$ and disrupts the symmetry of the spin-orbit beam shifts $Y_{\pm }$.

**Fig. 4.**
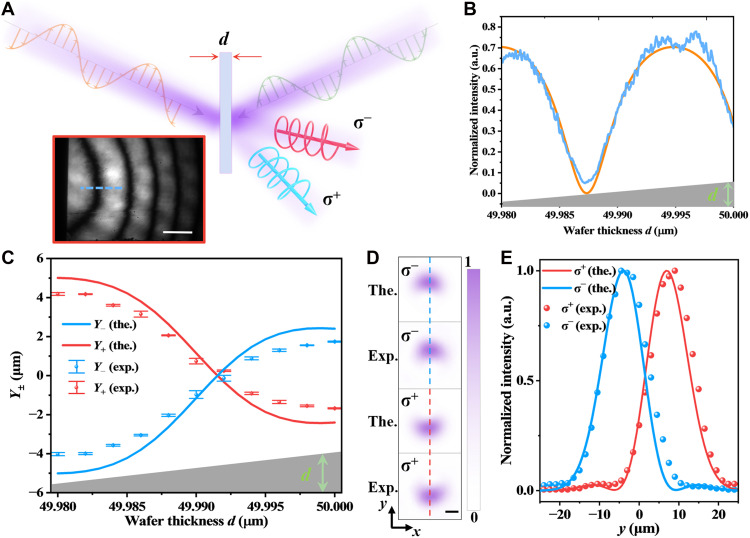
Spin-orbit beam shifts controlled by crystal thickness. (**A**) Schematic of coherent horizontally polarized beams incident onto an Si Wafer with variable thickness *d*. Inset: Fabry-Pérot fringes within Si wafer. Scale bar: 1 mm. (**B**) Normalized intensity profile along the dashed line in the inset of (A), along with the theoretical calculation. (**C**) Measured and theoretical spin-orbit beam shifts $Y_{\pm }$ versus wafer thickness *d*. The vertical error bars indicate the SE obtained from centroid extractions from independently recorded beam profile images (three measurements). (**D**) Spatial intensity distributions of σ^−^ and σ^+^ spin states at d=49.98 μm. Scale bar, 10 μm. (**E**) Corresponding cross-sectional intensity profiles along the dashed lines in (D).

Building on the search for tunable SOI responses, Fig. 4 explores the sensitivity of spin-orbit beam shifts to subtle variations in crystal thickness. As schematically shown in Fig. 4A, two coherent beams incident on the Si wafer with a variable optical path difference *d*. This configuration enables phase control through thickness modulation without altering the incidence angle or polarization state. The inset in Fig. 4A displays characteristic Fabry-Pérot interference fringes within the Si wafer, confirming high-quality internal reflection and enabling precise calibration of optical thickness. A line profile extracted along the blue dashed line (Fig. 4B) reveals sinusoidal fringe contrast, with the experimental data closely matching the theoretical calculation.

Within a narrow thickness variation of Δd=20 nm, a pronounced modulation of spin-orbit beam shifts is observed. As shown in Fig. 4C, beam shifts $Y_{\pm }$ vary as a function of *d*, under fixed incidence angle of $\theta_{1}=8.8{}^{\circ}$ and beam waist of $w_{0}=12\;\mu m$. The beam shifts for the σ^−^ and σ^+^ spin states are clearly separated and oppositely directed, demonstrating sensitivity to subwavelength-scale thickness variations. Figure 4D captures the spatial intensity distributions of two spin components at d=49.98 μm, where lateral spin separation is prominent. The corresponding intensity cross sections (Fig. 4E) along the dashed lines visually highlight the spin-orbit beam shifts, with consistent peak positions and linewidths between theory and experiment. These findings underscore the effectiveness of crystal thickness as a tunable parameter for active modulation of SOI-induced beam dynamics in a compact, all-optical platform.

### Ultrafast dynamic coherent controlled SOI

**Fig. 5.**
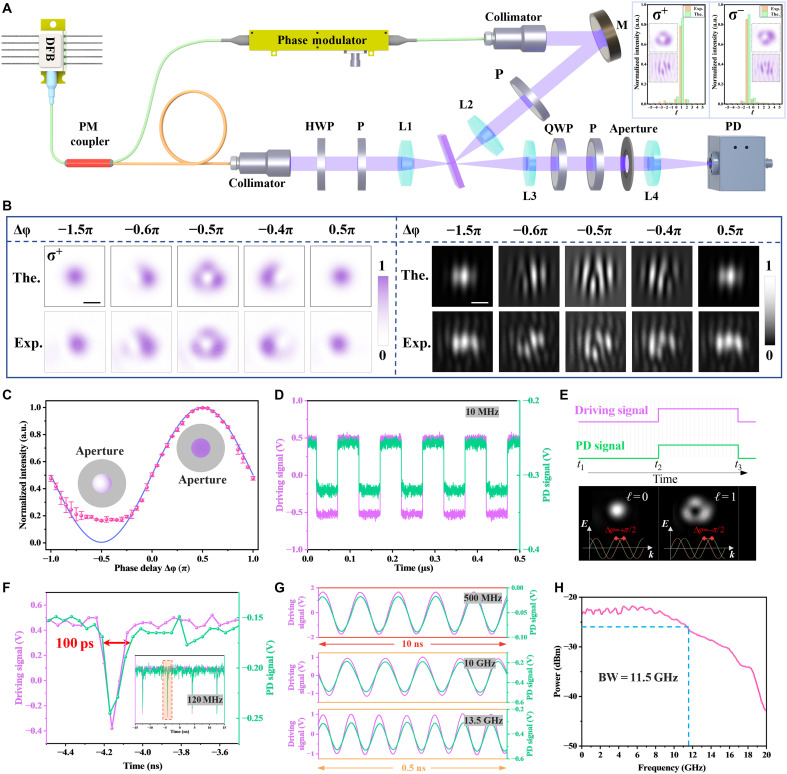
Dynamic coherent control of optical spin-orbit interactions. (**A**) Schematic of the experimental setup (see Materials and Methods for details). Insert of vortex-like intensity distributions, the interferograms, and the corresponding OAM spectra for the σ^−^ and σ^+^ spin states. (**B**) Simulated (top row) and experimental (bottom row) output intensity profiles (left columns) as a function of the relative phase delay Δφ. The right columns show the corresponding interferograms. Scale bars, 10 μm. (**C**) Measured normalized power versus Δφ. Insets illustrate aperture-filtered on-axis sampling. (**D**) Output response under 10-MHz square-wave phase modulation. (**E**) Schematic depiction of dynamic phase modulation governing transitions between Gaussian and vortex states. (**F**) Response of the system under 120-MHz periodic pulse modulation with 100-ps pulse width. The inset shows a stable pulse train. (**G**) Output response under sinusoidal phase modulation at 500 MHz, 10 GHz, and 13.5 GHz. (**H**) Frequency response of the system measured with a vector network analyzer, revealing a 3-dB bandwidth (BW) of ~11.5 GHz.

To experimentally implement dynamic coherent control of SOI, an optical setup as illustrated in Fig. 5A was constructed. A 1064-nm distributed-feedback (DFB) laser is equally split into signal and control arms via a 1 × 2 polarization-maintaining (PM) fiber coupler. The signal beam is collimated, linearly horizontally polarized, and focused to a 8-μm waist onto a Si wafer at $\theta_{1}=8.0{}^{\circ}$. The control beam, likewise horizontally polarized and spatially overlapped with the signal, undergoes phase modulation through a high-speed electro-optic phase modulator to precisely tune the phase delay Δφ. At the output, a quarter-wave plate (QWP) and Glan polarizer are used to project the σ^−^ and σ^+^ spin states, which are then imaged onto a charge-coupled device (CCD) camera.

As shown in the inset of Fig. 5A, both spin components exhibit vortex-like intensity distributions. The interferograms and OAM spectra confirm opposite topological charges for the σ^−^ and σ^+^ spin states. Figure 5B presents the evolution of output beam profiles with varying Δφ from −1.5 to +0.5π, highlighting the reversible transformation between Gaussian and vortex beams. The interferograms confirm the corresponding transitions of topological charge from ℓ = 0 to ℓ = +1 and back, verifying dynamic SOI modulation. Similar reversible transitions for the σ^−^ component, with the topological charge switching between ℓ = 0 and ℓ = −1 (fig. S6).

A circular aperture acts as a spatial filter to sample the on-axis (central) intensity, which provides an indirect yet robust indicator of the Gaussian-vortex transition (Fig. 5C). The normalized intensity exhibits sinusoidal dependence on Δφ, matching theoretical predictions, with maximum contrast at Δφ = −1.5π and +0.5π. The slight deviation from zero at the minima is attributed to diffraction-induced leakage through the aperture.

To evaluate the dynamic performance, a series of driving signals was applied to the electro-optic modulator. The transmitted intensity, filtered by an aperture and detected by a high-speed photodetector, was recorded via an oscilloscope. Under 10-MHz square-wave modulation (Fig. 5D), the output intensity exhibits clean and repeatable switching, consistent with spin-orbit transitions between Gaussian and vortex modes. Figure 5E schematically illustrates this dynamic modulation, where the topological structure of the output field toggles between ℓ = 0 and ℓ = ±1 as the phase delay crosses ±π/2. When driven by 120-MHz pulse signals with a pulse width of 100 ps (Fig. 5F), the system responds on a picosecond timescale, indicating ultrafast modulation capability. The inset of Fig. 5F further demonstrates stable operation under periodic pulsed excitation, confirming the robustness of the dynamic coherent control scheme.

Further extending to sinusoidal driving, Fig. 5G presents the photodetector responses under continuous-wave phase modulation at 500 MHz, 10 GHz, and 13.5 GHz, respectively. In all cases, the output closely follows the input signal, demonstrating coherent SOI modulation up to the gigahertz regime. The slight phase lag and amplitude distortion at higher frequencies are attributed to the finite electro-optic link bandwidth. To assess the full frequency response, the transfer function was measured using a vector network analyzer (Fig. 5H), revealing a 3-dB system bandwidth of ~11.5 GHz. Given the independently measured photodetector bandwidth (13.6 GHz; fig. S7) and the oscilloscope bandwidth (20 GHz), the electro-optic link is the dominant limitation for the system bandwidth and therefore sets the practical speed limit of our modulation experiment. Collectively, these results demonstrate that dynamic, high-speed, and reversible control of optical SOI can be achieved solely through phase modulation on a simple isotropic crystal platform, without altering polarization, incidence geometry, or material structure, thus providing a powerful and scalable approach to ultrafast spin-orbit photonics.

### Coherent control of dual-channel routing of spin-orbit state

**Fig. 6.**
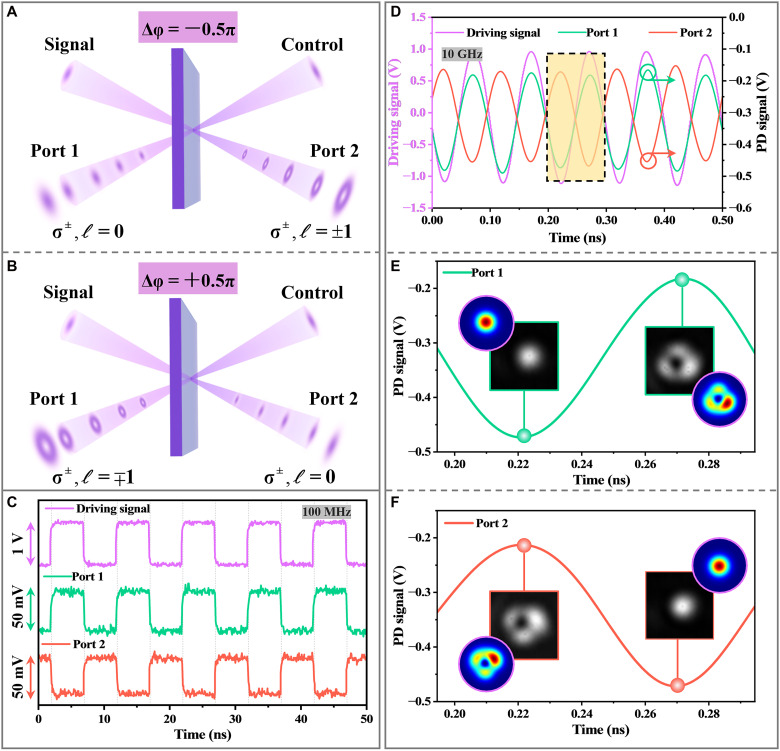
Dual-port coherent routing of spin-orbit optical states via phase-controlled modulation. (**A** and **B**) Schematic illustrations of spin-orbit output states under phase delays of Δφ=+π/2 (A) and Δφ=−π/2 (B), respectively. Under Δφ=+π/2, port 1 outputs a Gaussian beam (ℓ = 0), while port 2 emits a spin-dependent vortex beam (ℓ = ±1); reversing the phase delay swaps the output states at the two ports. (**C**) Output response under 100-MHz square-wave modulation, showing binary switching behavior with antiphase characteristics between ports 1 and 2. (**D**) Output response under 10-GHz sinusoidal modulation. (**E** and **F**) Zoomed-in PD signals at port 1 (E) and port 2 (F), with insets showing static CCD images of the output beams taken at the indicated phase delays.

To further demonstrate the real-time coherent control of SOI, Fig. 6 presents dynamic switching between output ports governed by the input phase delay. Specifically, Δφ is tuned to ±π/2, corresponding to two distinct spin-orbit configurations (see Materials and Methods for details). When the phase delay is set to Δφ=−π/2, as shown in Fig. 6A, port 1 outputs a Gaussian mode with no OAM, i.e., ℓ = 0, while port 2 emits a vortex beam carrying spin-dependent OAM with topological charge ℓ = ±1. In this case, the spin-OAM mapping occurs at port 2: The σ^+^ component carries ℓ = +1, and the σ^−^ component carries ℓ = −1. Conversely, when the phase delay is reversed to Δφ=+π/2 (Fig. 6B), the spatial profiles at the two ports are reversed. The vortex beam now emerges from port 1, while the Gaussian beam exits from port 2. The spin-OAM mapping also switches sides. At this setting, port 1 becomes the site of spin-orbit conversion, where σ^+^ corresponds to ℓ = −1 and the σ^−^ to ℓ = +1, effectively inverting the spin-OAM coupling compared to the −π/2 case. Note that Fig. 6 is presented in the circular basis (σ^±^) using a QWP and a polarizer. Without an analyzer, the output corresponds to a coherent superposition of σ^±^ components, and its polarization texture is governed by the relative amplitudes and phase of σ^±^ components. Full-Stokes parameter maps for both ports at Δφ = ±π/2 (figs. S3 and S4) directly confirm that the output beams form cylindrically polarized vector beams (linear polarized analyzation in movies S1 and S2), whose type (radial, azimuthal, etc.) also depends on Δφ.

The dynamic performance of the system under active phase control is experimentally demonstrated in Fig. 6C. When driven by a 100-MHz square-wave signal, the photodetector signals from port 1 and port 2 (σ^+^ components) show binary switching behavior with exact antiphase characteristics. This observation confirms the spin-dependent routing of Gaussian and vortex modes under alternating Δφ. When subjected to 10-GHz sinusoidal modulation (Fig. 6D), the output signals at both ports precisely follow the driving waveform, underscoring the ultrafast response of the system. Figure 6 (E and F) presents zoomed views of the photodetector responses for both ports, alongside the recorded static CCD images at fixed phase delays, illustrating the spatial beam profiles for the corresponding setpoints. The insertion loss of the dual-port router (Fig. 6) is 0.31 dB at Δφ = −π/2 and 0.29 dB at Δφ = +π/2 (fig. S8). The mode- and port-resolved contrast ratios are CR_ℓ,1_ = 18.06/12.08/13.88 dB for ℓ = 0/−1/+1 at port 1 and CR_ℓ,2_ = 17.90/13.76/13.37 dB at port 2. The corresponding port cross-talks are XT_ℓ_(+π/2) = −17.29/−12.82/−13.72 dB and XT_ℓ_(−π/2) = −17.10/−12.41/−13.33 dB for ℓ = 0/−1/+1, respectively (tables S1 and S2). Movie S3 show the beam profile of the port 2 σ^+^ component at Δφ = −π/2 remains essentially unchanged for 32 min, demonstrating robust long-timescale stability. Overall, such SOI-induced beam reconfiguration under high-speed phase modulation validates the potential of dynamic coherent control for ultrafast optical signal steering.

## DISCUSSION

To summarize, we demonstrate a gigahertz-level, reversible, and structurally passive scheme for dynamically reconfiguring optical SOI using phase-only coherent interference between two counter-propagating beams. While coherent control has been widely exploited to realize programmable multiport functionality in CPA/CPD/BIC-enabled platforms (*37*–*44*), our key distinction is that we harness coherent interference as a high-speed control knob to modulate planar-interface SOI in an unpatterned slab. This enables dynamic control of spin-dependent beam manipulation, including spin-orbit beams shifts, reversible transitions between Gaussian and spin-dependent vortex modes, and even spin-OAM states routing, without modifying the material structure or invoking any intrinsic anisotropy (*21*–*29*).

By tilting both the signal and control beams, a controllable azimuthal phase gradient is introduced, leading to spin-orbit beam shifts. Additional tunability is achieved by varying the input polarization angle α or introducing slight phase delay, enabling control of spin-dependent beam shifts and the spatial distribution of spin-resolved fields. Notably, giant spin-orbit beam shifts reaching up to ~10λ are observed between the $\sigma^{+}$ and $\sigma^{-}$ components. Furthermore, we find that even a 20-nm variation in crystal thickness can substantially modulate the spin-orbit beam shifts, establishing the wafer thickness as an effective static tuning parameter that expands the control landscape.

A central outcome of our work is the experimental realization of coherent transitions between spin-dependent Gaussian and vortex beams, governed solely by the relative phase of two counter-propagating input beams. Tuning the relative phase from +π/2 to −π/2 results in transition of the output topological charge from ℓ = 0 and ℓ = ±1 for opposite spin components. Such functionality is rarely accessible in conventional spin-orbit systems, where the spin-orbit response is predefined by material geometry or anisotropy (*20*–*29*).

The demonstrated coherent SOI control substantially outperforms previous reconfigurable spin-orbit systems in terms of modulation speed (*21*–*30*). Prior approaches based on liquid crystals (*23*–*26*), phase-change materials (*28*, *29*), or metasurfaces (*27*) are fundamentally limited by slow material responses, restricting operation to kilohertz to megahertz range.

In contrast, our system achieves modulation bandwidths up to 11.5 GHz, as verified by network analyzer measurements, marking a key advancement toward gigahertz-level SOI-enabled optical logic, switching, and dynamic modulation platforms.

In the broader landscape of OAM switching, electro-optically driven q-plate architectures can reverse the sign of a predefined OAM mode with potentially gigahertz-class operation, yet they typically toggle between two fixed vortex states (*47*–*49*). Other platforms, such as vortex microlasers (*31*, *50*–*52*) and dynamic meta-optical systems (*53*, *54*), have already demonstrated ultrafast (picosecond-scale) OAM switching via different mechanisms. In contrast, by coherently controlling planar-interface SOI in an unpatterned isotropic slab, we realize gigahertz-level, reversible, and phase-controlled mode transitions from a Gaussian mode (ℓ = 0) to a first-order OAM vortex mode (ℓ = ±1), with the sign of the topological charge determined by the spin state.

Note that high-speed modulation traces in Fig. 5 are obtained from the aperture-filtered on-axis intensity rather than spatially resolved ultrafast imaging of the full beam profile. Advanced ultrafast imaging schemes, such as compressed ultrafast photography (*55*, *56*), all-optical burst/framing approaches (*57*, *58*), chirped spectral mapping (*59*), and spectrum-circuit approaches (*60*), could be adapted to offer spatially resolved confirmation of mode transitions under high-speed phase modulation in our platform.

Moreover, our system demonstrates dual-port coherent routing of spin-OAM states (Fig. 6), where the output port of a given spin-OAM state is dynamically determined by the sign of the phase delay. This dual-port architecture opens avenues in spin-based signal demultiplexing, structured light communication, and photonic switching, allowing simultaneous and reversible encoding of spin and orbital degrees of freedom.

## MATERIALS AND METHODS

### Optical setup

The experimental setup consists of a dual-beam configuration enabling coherent control of spin-orbit states (Fig. 5A). A continuous-wave DFB laser (Aerodiode1064LD-2b-LN-1) centered at 1064 nm is coupled into a PM fiber and split equally by a 1 × 2 PM fiber coupler (Lbtek PMC-1064-50-FA) into signal and control beams. The control beam passes through an electro-optic phase modulator (Conquer KG-PM-10-10G-PP-FA) to introduce a tunable phase delay Δφ. Two beams are collimated by fiber collimators (Thorlabs F220APC-1064). In the signal arm, a half-wave plate (Lbtek HWP20-1064BT) and a Glan-Taylor polarizer (P, Thorlabs GT10-B) are used to control the beam power and polarization angle α. The collimated beam is reflected by a mirror (M, Thorlabs BB2-E03), mounted on a motorized rotation stage to allow precise adjustment of the incident angle, and then passes through a polarizer to select the desired polarization. Two beams are focused by plano-convex lenses (L1/L2, *f* = 30 mm, Lbtek MBCX10307-B) onto a double-side polished p-type Si wafer (100-oriented), forming spatially overlapped counter-propagating beams. Except for Fig. 4 (where *d* is scanned), all experiments and calculations use *d* = 49.99 μm for λ = 1064 nm.

To resolve spin states (σ^+^ or σ^−^), a QWP (Lbtek QWP10-1064B), along with a Glan-Taylor polarizer, is inserted in the output arms. For spatial characterization, the output is relayed to a CCD camera (Daheng ME2P-1230-23U3M) via an imaging lens (L3, *f* = 150 mm, Lbtek MBCX106157-B). Alternatively, for intensity-based measurements, an aperture is used to isolate the central region of interest, and the transmitted light is coupled into a photodetector (New Focus 1554-B-50) for temporal signal acquisition on an oscilloscope (Tektronix DSA72004B Digital Serial Analyzer, 20 GHz, 50 GS/s, 4 Ch). The driving signals of phase modulation are generated from either an arbitrary waveform generator (Tektronix AWG7122B) or a vector network analyzer (Agilent N5222A), the latter also used for 3-dB bandwidth characterization.

### Angular response for plane wave incidence

A monochromatic plane wave (1064 nm) is incident from air (*n*_0_ = 1) onto a Si wafer at an angle θ_1_. The complex refractive index of Si is written as $n=n^{'}+\mathrm{i\kappa}$. For compact analytical derivations, we take the lossless limit (κ → 0), while the full complex index is used in all numerical calculations throughout the manuscript. At 1064 nm, κ = 8.26 × 10^−5^, corresponding to an intensity attenuation coefficient ξ = 4πκ/λ ≈ 9.76 × 10^2^ m^−1^. For a standard 49.99-μm slab, the single-pass absorption is ~4.8%. The refracted angle inside the wafer, θ_2_, is determined by Snell’s law as $\theta_{2}=\text{asin}\left(\text{sin}\theta_{1}/n\right)$. The optical response at the air-silicon interface is characterized by the Fresnel reflection and transmission coefficients

$$\begin{array}{c} r_{s}^{\text{intf}}=\frac{\text{cos}\theta_{1}-n\text{cos}\theta_{2}}{\text{cos}\theta_{1}+n\text{cos}\theta_{2}},r_{p}^{\text{intf}}=\frac{n\text{cos}\theta_{1}-\text{cos}\theta_{2}}{n\text{cos}\theta_{1}+\text{cos}\theta_{2}},\\ t_{s}^{\text{intf}}=\frac{2\text{cos}\theta_{1}}{\text{cos}\theta_{1}+n\text{cos}\theta_{2}},t_{p}^{\text{intf}}=\frac{2\text{cos}\theta_{1}}{n\text{cos}\theta_{1}+\text{cos}\theta_{2}} \end{array}$$ (5)

which depend on the polarization and incident angle. With small angle approximation and a sufficiently high refractive index *n*, the interface behaves nearly as normal incidence, which gives $r_{p}^{\text{intf}}\approx -r_{s}^{\text{intf}}$, induced by the π phase shift for vertical polarization wave when reflecting from a higher-index medium. Within the wafer, light undergoes multiple internal reflections, each round trip contributing a cumulative phase of $\delta =2\mathrm{\pi n}\text{cos}\theta_{2}d/\lambda$, where *d* is the wafer thickness. Multiple reflections within the wafer lead to Fabry-Pérot–type interference, which modifies the overall amplitude and phase of the reflected and transmitted waves. The modified effective coefficients are given by

$$r_{p,s}=\frac{-2{\mathrm{ie}}^{i\delta }\text{sin}{\delta r}_{p,s}^{\text{intf}}}{1-{\left(r_{p,s}^{\text{intf}}\right)}^{2}e^{i2\delta }},t_{p,s}=\frac{{\left(t_{p,s}^{\text{intf}}\right)}^{2}e^{i\delta }}{1-{\left(r_{p,s}^{\text{intf}}\right)}^{2}e^{i2\delta }}$$ (6)

which incorporate both the intrinsic interfacial Fresnel response and the cumulative phase acquired during propagation within the wafer. The phase of complex coefficients $r_{p,s}$ are related to the sign of $r_{p,s}^{\text{intf}}$. In addition, *t_p_* and *r_p_* have a phase difference of π/2, whereas *t_s_* and *r_s_* differ in phase by −π/2. In contrast, *t_p_* and *t_s_* remain phase aligned.

In the coherent dual-beam configuration, the total output wave at the front and rear surfaces of the wafer arises from the superposition of forward and backward propagating components, each modulated by the Fresnel reflection and transmission responses. For each polarization state, the field amplitudes can be described as $B_{1,2}^{p,s}=r_{p,s}A_{1,2}^{p,s}+t_{p,s}A_{2,1}^{p,s}$, indicating that constructive or destructive interference can be engineered at the outputs depending on the Fresnel responses and the relative phase delay between two counter-propagating waves. Specifically, assuming both incident beams are of unit amplitude and have a relative phase delay of −π/2, the interference at the outputs are only determined by the Fresnel response. When the modified reflected and transmitted coefficients are of equal amplitude, the two counter-propagating waves interfere resulting in polarization-selective outputs: $|B_{1}^{p}|=2|r_{p}|$, $|B_{1}^{s}|=0$, $|B_{2}^{p}|=0$, and $|B_{2}^{s}|=2|r_{s}|$. For another case of Δφ=+π/2, the output ports of constructive and destructive interference are reversed. The condition of coherent extinction leads to a closed-form expression for the critical cumulative phase $\Delta_{p,s}$, which corresponds to the cumulative phase δ and adheres the constraint imposed by the Fresnel response as $\text{sin}\Delta_{p,s}={\left(-1\right)}^{j}\left[1-{\left(r_{p,s}^{\text{intf}}\right)}^{2}\right]/2r_{p,s}^{\text{intf}}$. Constructive or destructive interference arises at angles where the cumulative phase satisfies $\delta =\Delta_{p,s}$.

### Derivation of the scattering matrix

We first establish the laboratory Cartesian frame (*x*, *y*, and *z*), with the *z* axis that is normal to the Si wafer. The coordinate frames (*x_a_*, *y_a_*, and *z_a_*) denote the central wave vectors of incidence, reflection, and transmission, respectively, with *a* labeled as *a* = *i*, *r*, *t*. For a paraxial incident beam with an angular spectrum of $\tilde{A}={\tilde{A}}_{0}\left[b{\hat{e}}_{x}+c{\hat{e}}_{y}\right]$, where *b* and *c* are the complex coefficients for horizontal and vertical polarization states, respectively, and the Gaussian profile is ${\tilde{A}}_{0}=\frac{w_{0}}{\sqrt{2\pi }}\text{exp}\left[-{\omega_{0}}^{2}\left({k_{x}}^{2}+{k_{y}}^{2}\right)/4\right]$. The transformation from the local coordinate frame to a spherical global coordinate frame is achieved using a sequence of rotation operations $\hat{U}\left(\theta_{a},\kappa_{x},\kappa_{y}\right)={\hat{R}}_{y}\left(\theta_{a}+\kappa_{x}\right){\hat{R}}_{z}\left(\kappa_{y}/\text{sin}\theta_{a}\right){\hat{R}}_{y}\left(-\theta_{a}\right)$, where the rotation matrices ${\hat{R}}_{y}$ and ${\hat{R}}_{z}$ align the local beam frame to the global spherical coordinates. Under the paraxial approximation, this transformation reduces to

$$\hat{U}\left(\theta_{a},\kappa_{x},\kappa_{y}\right)\approx \left[\begin{array}{cc} 1 & \kappa_{y}/\text{sin}\theta_{a}\\ -\kappa_{y}/\text{sin}\theta_{a} & 1 \end{array}\right]$$ (7)

The transmitted angular spectrum is connected with the incident one via a Fresnel matrix ${\hat{F}}_{a}$, which, in the paraxial limit, depends solely on the angle of incidence

$${\hat{F}}_{a}\approx \left[\begin{array}{cc} f_{p}^{a}+\kappa_{x}\partial f_{p}^{a}/\partial \theta & 0\\ 0 & f_{s}^{a}+\kappa_{x}\partial f_{s}^{a}/\partial \theta \end{array}\right]$$ (8)

Here, $f_{p,s}^{a}$ are the Fresnel reflection/transmission coefficients for horizontally and vertically polarized components at interface *a*.

To connect the angular spectrum of the transmitted local field with the incident one, we use

$$\tilde{B}={\hat{U}}^{+}{\hat{F}}_{a}\hat{U}\tilde{A}$$ (9)

We then define a generalized transformation matrix ${\hat{T}}_{a}={\hat{U}}^{+}\left(\theta_{a},\kappa_{x},\kappa_{y}\right){\hat{F}}_{a}\hat{U}\left(\theta ,\kappa_{x},\kappa_{y}\right)$, which, in the linear polarization basis, can be written explicitly as

$${\hat{T}}_{a}\approx \left[\begin{array}{cc} f_{p}^{a}+\kappa_{x}\partial f_{p}^{a}/\partial \theta_{1} & \kappa_{y}\left[\text{cos}\theta_{1}f_{p}^{a}-\text{cos}\theta_{a}f_{s}^{a}\right]/\text{sin}\theta_{1}\\ -\kappa_{y}\left[\text{cos}\theta_{1}f_{p}^{a}-\text{cos}\theta_{a}f_{s}^{a}\right]/\text{sin}\theta & f_{s}^{a}+\kappa_{x}\partial f_{s}^{a}/\partial \theta_{1} \end{array}\right]$$ (10)

where θ_1_ is the incident angle. In our experiments (λ=1064 nm) the focused Gaussian spot has $w_{0}$ =12 μm, corresponding to $\text{NA}\approx \lambda /\left(\pi w_{0}\right)=0.028$ (i.e., $k_{⊥}/k_{0}\leq 0.028$), which well satisfies the paraxial condition underlying Eqs. 7 to 10.

By applying the transformation matrix to the forward and backward propagating beams across the Si wafer, the transmitted and reflected fields on each side of the wafer can be expressed as

$$\left[\begin{array}{c} {\tilde{B}}_{1}^{p}\\ {\tilde{B}}_{1}^{s} \end{array}\right]=\left[\begin{array}{cc} r_{p}+\kappa_{x}r_{p}^{\prime } & \kappa_{y}N\\ -\kappa_{y}N & r_{s}+\kappa_{x}r_{s}^{\prime } \end{array}\right]\left[\begin{array}{c} {\tilde{A}}_{1}^{p}\\ {\tilde{A}}_{1}^{s} \end{array}\right]$$ (11A)

$$\left[\begin{array}{c} {\tilde{B}}_{1}^{p}\\ {\tilde{B}}_{1}^{s} \end{array}\right]=\left[\begin{array}{cc} t_{p}+\kappa_{x}t_{p}^{\prime } & \kappa_{y}M\\ \kappa_{y}M & t_{s}+\kappa_{x}t_{s}^{\prime } \end{array}\right]\left[\begin{array}{c} {\tilde{A}}_{2}^{p}\\ {\tilde{A}}_{2}^{s} \end{array}\right]$$ (11B)

$$\left[\begin{array}{c} {\tilde{B}}_{2}^{p}\\ {\tilde{B}}_{2}^{s} \end{array}\right]=\left[\begin{array}{cc} t_{p}+\kappa_{x}t_{p}^{\prime } & \kappa_{y}M\\ \kappa_{y}M & t_{s}+\kappa_{x}t_{s}^{\prime } \end{array}\right]\left[\begin{array}{c} {\tilde{A}}_{1}^{p}\\ {\tilde{A}}_{1}^{s} \end{array}\right]$$ (11C)

$$\left[\begin{array}{c} {\tilde{B}}_{2}^{p}\\ {\tilde{B}}_{2}^{s} \end{array}\right]=\left[\begin{array}{cc} r_{p}+\kappa_{x}r_{p}^{\prime } & \kappa_{y}N\\ -\kappa_{y}N & r_{p}+\kappa_{x}r_{s}^{\prime } \end{array}\right]\left[\begin{array}{c} {\tilde{A}}_{2}^{p}\\ {\tilde{A}}_{2}^{s} \end{array}\right]$$ (11D)

where $M=\left(r_{p}+r_{s}\right)\text{cot}\theta_{1}$ and $N=\left(t_{p}-t_{s}\right)\text{cot}\theta_{1}$. Combining the above relations, we obtain the complete scattering matrix **S** for the coherent dual-beam configuration, relating all input and output angular spectral components

$$S=\left[\begin{array}{cccc} r_{p}+\kappa_{x}r_{p}^{\prime } & t_{p}+\kappa_{x}t_{p}^{\prime } & \kappa_{y}M & \kappa_{y}N\\ t_{p}+\kappa_{x}t_{p}^{\prime } & r_{p}+\kappa_{x}r_{p}^{\prime } & \kappa_{y}N & \kappa_{y}M\\ -\kappa_{y}M & \kappa_{y}N & r_{s}+\kappa_{x}r_{s}^{\prime } & t_{s}+\kappa_{x}t_{s}^{\prime }\\ \kappa_{y}N & -\kappa_{y}M & t_{s}+\kappa_{x}t_{s}^{\prime } & r_{s}+\kappa_{x}r_{s}^{\prime } \end{array}\right]$$ (12)

### SOI-induced optical spatial evolution

On the basis of the scattering relation of $\tilde{B}=S\tilde{A}$, under coherent dual-beam incidence (e.g., horizontally polarized beams), the angular spectra at two output ports are expressed in the spin basis as

$$\begin{array}{c} {\tilde{B}}_{1}={\tilde{A}}_{0}\left\{\left(r_{p}+t_{p}e^{i\Delta \varphi }\right)+\kappa_{x}\left(r_{p}^{\prime }+t_{p}^{\prime }e^{i\Delta \varphi }\right)-i{\sigma \kappa }_{y}\left(-M+{\mathrm{Ne}}^{i\Delta \varphi }\right)\right\}{\hat{e}}_{\sigma }\\ {\tilde{B}}_{2}={\tilde{A}}_{0}\left\{\left(r_{p}e^{i\Delta \varphi }+t_{p}\right)+\kappa_{x}\left(r_{p}^{\prime }e^{i\Delta \varphi }+t_{p}^{\prime }\right)-i{\sigma \kappa }_{y}\left(-{\mathrm{Me}}^{i\Delta \varphi }+N\right)\right\}{\hat{e}}_{\sigma } \end{array}$$ (13)

The optical shifts ($X_{\pm }$) and spin-orbit shifts ($Y_{\pm }$) are thus obtained

$$X_{\pm }=\frac{\int {\tilde{B}}_{j}^{\sigma }\partial {\tilde{B}}_{j}^{\sigma }/\partial k_{x}dk_{x}{\mathrm{dk}}_{y}}{\int {|{\tilde{B}}_{j}^{\sigma }|}^{2}dk_{x}{\mathrm{dk}}_{y}},Y_{\pm }=\frac{\int {\tilde{B}}_{j}^{\sigma }\partial {\tilde{B}}_{j}^{\sigma }/\partial k_{y}dk_{x}{\mathrm{dk}}_{y}}{\int {|{\tilde{B}}_{j}^{\sigma }|}^{2}dk_{x}{\mathrm{dk}}_{y}}$$ (14)

In our experiment, beam centroid positions were extracted from the recorded CCD beam profiles using an intensity-weighted centroid algorithm. For each parameter point in Figs. 2 to 4, we repeated the measurement three times; the reported centroid shifts are the mean values, and the error bars denote SE across repeats.

For port 2, when the effective total field amplitude ($r_{p}e^{i\Delta \varphi }+t_{p}$) is not close to zero, namely, the weak interaction regime, the output field can be approximated as ${\tilde{B}}_{2}\approx {\tilde{A}}_{\text{eff}}\cdot \left[e^{-i{\sigma \gamma \kappa }_{y}}+{\beta \kappa }_{x}\right]{\hat{e}}_{\sigma }$, with the effective amplitude defined as A˜eff=A˜(rpeiΔφ+tp)0. The SOI strength is characterized by the dimensionless complex coupling coefficient $\gamma =\frac{-{\mathrm{Me}}^{i\Delta \varphi }+N}{r_{p}e^{i\Delta \varphi }+t_{p}}$, while the term $\beta =\frac{r_{p}^{\prime }e^{i\Delta \varphi }+t_{p}^{\prime }}{r_{p}e^{i\Delta \varphi }+t_{p}}$ accounts for the angular dispersion of the Fresnel coefficients.

The complex coupling phase $-{\sigma \gamma \kappa }_{y}$, introduced along the transverse wave vector direction *k_y_*, directly governs the SOI of the output field. The spin-orbit beam shifts are jointly modulated by both the incident angle and the phase delay. In contrast, the in-plane phase term β, originating from the angular dispersion of the Fresnel coefficients, remains spin independent. If the polarization symmetry is broken, then the spin-independent term β lifts the spin degeneracy, enabling spin-selective control.

In particular, by setting relative phase Δφ=−π/2, when the Fresnel coefficients satisfy the condition $t_{p}-{\mathrm{ir}}_{p}=0$, strong SOI arises. In this condition, the complex coefficients of the transverse components $\kappa_{x,y}$, namely, $\left(-ir_{p}^{\prime }+t_{p}^{\prime }\right)$ and (iM+N), have a relative phase difference of π/2. Thus, the output field takes the form of ${\tilde{B}}_{2}={\tilde{A}}_{0}\left\{\kappa_{x}|-ir_{p}^{\prime }+t_{p}^{\prime }|-i{\sigma \kappa }_{y}|\mathrm{iM}+N|\right\}{\hat{e}}_{\sigma }$, where the complex phase structure leads to opposite topological vortex phases being encoded in the output σ^−^ and σ^+^ spin states. This spin-dependent phase structure becomes particularly evident when the orthogonal transverse components are of comparable amplitude.

### Dual-channel routing of spin-orbit states

To elucidate the spin-orbit routing under coherent dual-beam incidence, we consider two representative cases of the relative phase delay between the incident beams, namely, Δφ=±π/2. This phase offset plays a critical role in determining the spatial mode profiles and topological charge carried by the output field.

First, for the case of Δφ=−π/2, when $t_{p}-{\mathrm{ir}}_{p}=0$, the $\kappa_{x}$-related terms dominate in ${\tilde{B}}_{1}$ due to the strong in-plane momentum component, i.e., $|r_{p}^{\prime }-it_{p}^{\prime }|\gg |r_{p}-{\mathrm{it}}_{p}|$ and $|r_{p}^{\prime }-it_{p}^{\prime }|\gg |-M-\text{iN}|$. The complex coefficients of the $\kappa_{x,y}$ components, $\left(-ir_{p}^{\prime }+t_{p}^{\prime }\right)$ and (iM+N), have a phase difference of π/2. Hence, the fields simplify to

$$\begin{array}{c} {\tilde{B}}_{1}\approx {\tilde{A}}_{0}\left\{\kappa_{x}\left(r_{p}^{\prime }-it_{p}^{\prime }\right)\right\}{\hat{e}}_{\sigma }\\ {\tilde{B}}_{2}={\tilde{A}}_{0}\left\{|-ir_{p}^{\prime }+t_{p}^{\prime }|\kappa_{x}-\mathrm{i\sigma}|\mathrm{iM}+N|\kappa_{y}\right\}{\hat{e}}_{\sigma } \end{array}$$ (15)

It is evident that ${\tilde{B}}_{1}$ corresponds to a spatially shifted Gaussian beam without spin-orbit coupling, whereas ${\tilde{B}}_{2}$ carries a spin-dependent first-order vortex phase.

In the opposite case where Δφ=+π/2, when $r_{p}+{\mathrm{it}}_{p}=0$, the inequality of the coefficients ($|ir_{p}^{\prime }+t_{p}^{\prime }|\gg |{\mathrm{ir}}_{p}+t_{p}|$ and $|ir_{p}^{\prime }+t_{p}^{\prime }|\gg |-\mathrm{iM}+N|$) leads to the dominance of the $\kappa_{x}$ term in ${\tilde{B}}_{2}$. In addition, the complex coefficients of the $\kappa_{x,y}$ components, $\left(r_{p}^{\prime }+it_{p}^{\prime }\right)$ and (−M+iN), have a phase difference of −π/2. Thus, the output fields can be simplified to

$$\begin{array}{c} {\tilde{B}}_{1}={\tilde{A}}_{0}\left\{|r_{p}^{\prime }+it_{p}^{\prime }|\kappa_{x}+\mathrm{i\sigma}|-M+\text{iN}|\kappa_{y}\right\}{\hat{e}}_{\sigma }\\ {\tilde{B}}_{2}\approx {\tilde{A}}_{0}\left\{\kappa_{x}\left(ir_{p}^{\prime }+t_{p}^{\prime }\right)\right\}{\hat{e}}_{\sigma } \end{array}$$ (16)

We observe that ${\tilde{B}}_{2}$ now forms the spatially shifted Gaussian mode, while ${\tilde{B}}_{1}$ carries the spin-dependent vortex phase with an opposite topological charge compared to the previous case. These two scenarios clearly demonstrate that spin-orbit states can be actively routed into two spatially separated output channels by simply tuning the relative phase difference between the two counter-propagating incident beams. The orthogonality of the Gaussian and vortex-like output modes allows for spin-selective spatial demultiplexing, where each spin state (σ^−^ and σ^+^) can be mapped to distinct spatial or modal channels, carrying opposite topological vortex phases.
