# Supplementary material for: Coherent control of optical spin-orbit interactions
Source: Sci Adv. 2026 Apr 10;12(15):eaec4604. doi: 10.1126/sciadv.aec4604 (PMC13068057; doi:10.1126/sciadv.aec4604)
Supplement: Supplementary file 1 — Supplementary Text Figs. S1 to S8 Tables S1 and S2 Legends for movies S1 to S3 [file sciadv.aec4604_sm.pdf]

Science Advances
AAAS

Supplementary Materials for

Coherent control of optical spin-orbit interactions

Hongwei Yang *et al.*

Corresponding author: Wenguo Zhu, zhuwg88@163.com

Sci. Adv. **12**, eaec4604 (2026)
DOI: 10.1126/sciadv.aec4604

The PDF file includes:

Supplementary Text
Figs. S1 to S8
Tables S1 and S2
Legends for movies S1 to S3

Other Supplementary Material for this manuscript includes the following:

Movies S1 to S3

Supplementary Text

When $n = n' + i\kappa$ is used, the propagation phase becomes complex, the propagation phase becomes complex:

$$\tilde{\delta} = \frac{2\pi}{\lambda}(n + i\kappa)d\cos\,\theta_2 = \delta + i\delta_i,$$

so the round-trip factor in Eq. 6 is modified by a deterministic exponential attenuation:

$$e^{i\tilde{\delta}} = e^{i\delta}e^{-\delta_i}.$$

Physically, κ introduces deterministic exponential attenuation of higher-order internal reflections; it does not destroy coherence, but reduces Fabry–Pérot fringe visibility/broadens resonances and limits the achievable extinction depth. In practice, finite absorption in Si slightly reduces the internally transmitted contribution ($|t|$) more strongly than $|r|$, which shifts the balance condition $|t|\approx|r|$ and makes the cancellation minimum finite, $|B_{\min}|^2 \sim (|t| - |r|)^2 \neq 0$. This incomplete cancellation leaves a small residual non-vortex (Gaussian-like) background that can interfere with the spin-dependent vortex term, producing a weak left–right imbalance in the horizontal polarization component and a slight distortion of the selected $\sigma\pm$ vortex-ring profile at 1064 nm.

Figure. S1 provides a wavelength cross-check to verify that the small output asymmetries observed at 1064 nm are absorption-limited rather than originating from an omission in the coherent-interference model. Under the same geometry and the same phase setpoint ($\Delta\varphi = -\pi/2$), the 1064-nm case shows a weak left–right imbalance in the horizontal-polarization intensity map I_H and a slightly distorted σ^+ vortex profile. This behavior is consistent with finite absorption in Si attenuating higher-order Fabry–Pérot contributions, thereby reducing the extinction depth and leaving a residual non-vortex background that interferes with the vortex component. In contrast, at 1550 nm (where Si absorption is substantially reduced), both the calculated and measured patterns become noticeably more symmetric for I_H and for the σ^+ vortex ring, approaching the lossless ideal. This wavelength dependence supports our interpretation that κ primarily limits the Fabry–Pérot fringe visibility/extinction depth, while the coherent-control routing mechanism remains unchanged.

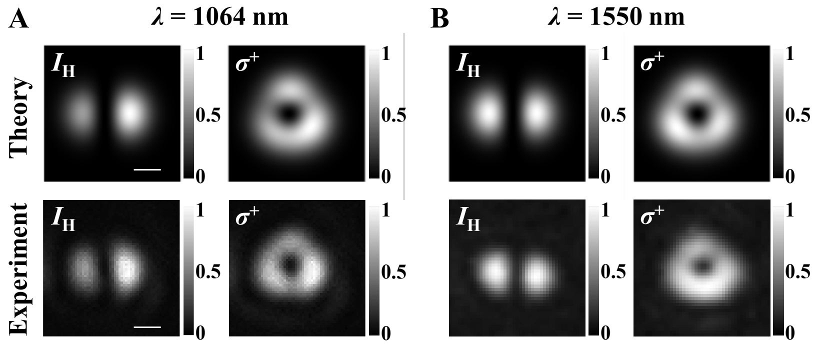

Fig. S1. Wavelength dependence of absorption-limited asymmetry (1064 nm vs 1550 nm). Comparison between theory (top row) and experiment (bottom row) for the output intensity distributions at (**A**) $\boldsymbol{\lambda = 1064}$nm with d = 49.99 μm, and (**B**) $\boldsymbol{\lambda = 1550}$nm under identical incident condition (θ_1=8°) and the same phase setpoint ($\boldsymbol{\Delta\varphi = -\pi/2}$) with d = 49.887 μm. For

each wavelength, the left panel shows the horizontal-polarization intensity map $\boldsymbol{I_H}$, and the right panel shows the intensity of the $\boldsymbol{\sigma}^+$component. Scalebar: 5 μm.

To directly verify robustness against slow environmental perturbations, we continuously record the Port-2 σ^+ beam profile (see Movie S1) at the routing setpoint ($\Delta\varphi$ fixed at −π/2). The recorded pattern remains essentially unchanged for >32 min under ambient laboratory conditions (no active feedback), demonstrating stable long-timescale operation at a fixed setpoint.

In addition, we measured the horizontal-polarization output beam profile at Port 2 while sweeping θ_1 across a narrow range (7.2°–9.0°) at the same fixed phase setpoint. As shown in Fig. S6, the horizontal component exhibits a distinctive and repeatable evolution versus θ_1 (including a clear extinction feature near the coherent-extinction angle), providing a practical "fingerprint" to confirm that the system remains at the intended operating condition during θ_1 sweeps and alignment checks.

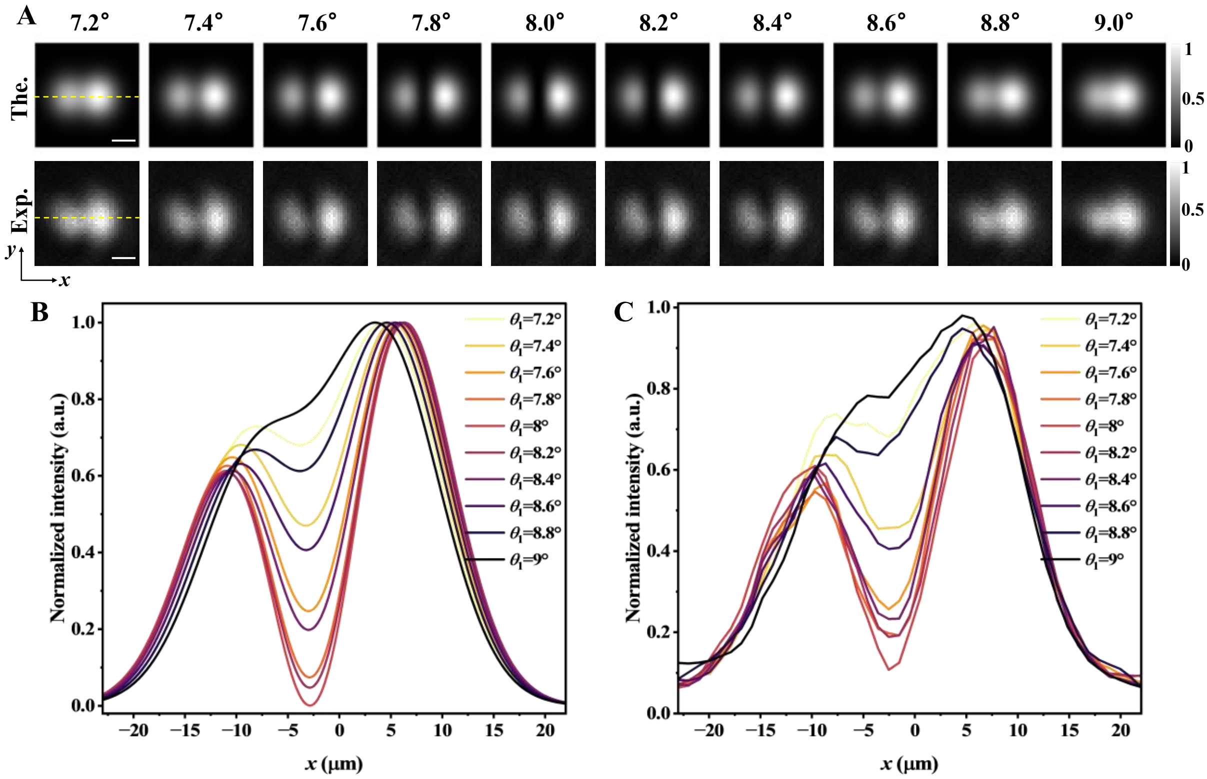

Fig. S2. Horizontal-polarization output beam profiles at Port 2 versus incidence angle θ_1 (robustness "fingerprint"). A Theoretical (top row) and experimental (bottom row) horizontal-polarization intensity patterns at Port 2 measured while sweeping θ_1 from 7.2° to 9.0° at a fixed coherent-interference setpoint ($\Delta\varphi$ = −π/2). **B, C** Corresponding normalized cross-sectional intensity profiles extracted along the dashed line in (**A**): (**B**) theory and (**C**) experiment. A pronounced extinction in the horizontal component appears near the coherent-extinction condition (around $\theta_1 \approx 8°$), consistent with destructive interference in the horizontally polarized output at Port 2. Scalebar: 10 μm.

To remove any ambiguity associated with polarization mixing or analyzer-dependent artifacts, we perform spatially resolved full-Stokes polarimetry across the complete output beam profiles at both output ports.

Experimentally, we record six analyzer images over the same field of view: linear bases (I_H, I_V, I_{+45}, I_{-45}) and circular bases (I_R, I_L). The Stokes parameters are reconstructed pixel-wise as (*46*):

$$S_0 = I_H + I_V, S_1 = I_H - I_V, S_2 = I_{+45} - I_{-45}, S_3 = I_R - I_L,$$

and the normalized maps S_1/S_0, S_2/S_0, and $S_3/S_0$to visualize the local polarization texture are show in Fig. S2-3. The measured Stokes maps are compared to the theoretical predictions calculated from the same model used in the main text. In both wavelength cases (1064 and 1550 nm), the reconstructed S_3/S_0maps exhibit a robust, spatially structured helicity distribution that matches theory at each port, confirming that the observed spin-dependent spatial separation is genuinely spin resolved rather than an artifact of projection optics. Under our 1064-nm experimental parameters, the Fresnel-weighted prefactors of the κ_x- and κ_y-related terms are generally unequal, causing the generated vortices deviate from an ideal pure OAM eigenstate and exhibit residual OAM sidebands.

Without an analyzer (S_0), the output corresponds to a coherent superposition of $\sigma^{\pm}$ components, and its polarization texture is governed by the relative amplitudes and phase of $\sigma^{\pm}$ components. Consistent with this picture, full-Stokes parameter maps for both ports at $\Delta\varphi = \pm\pi/2$ (Fig. S3 and S4) directly confirm directly confirm that the output beam form a cylindrically polarized vector beam (linear polarized analyzation in Movie S1 and S2), whose type (radial, azimuthal, etc.) also depends on $\Delta\varphi$.

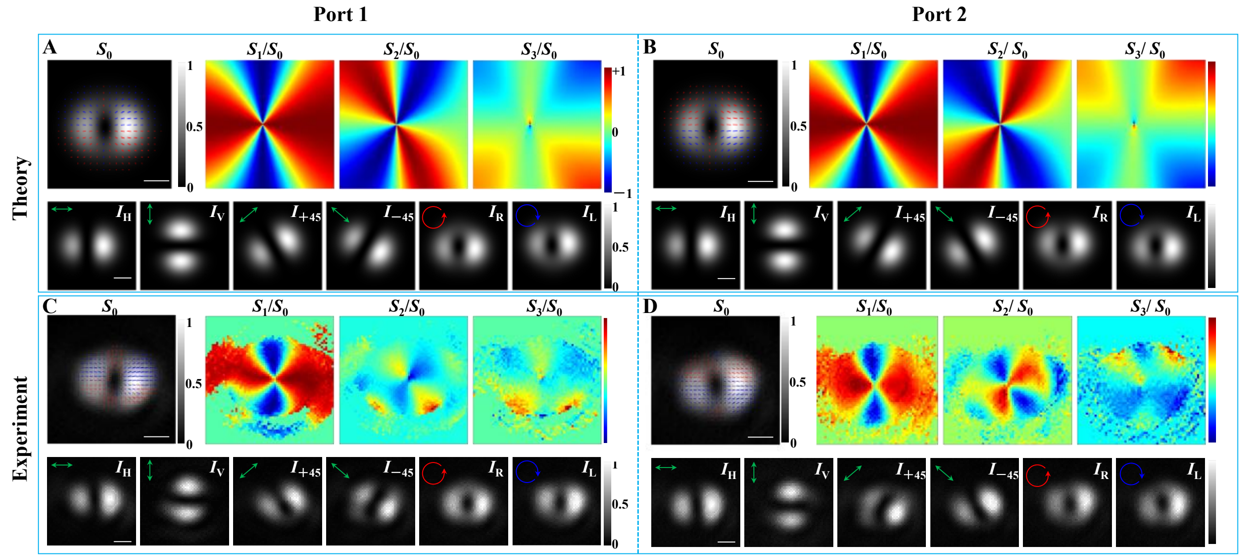

Fig. S3. Full-Stokes parameter maps across the output beam profiles at 1064 nm.

A, B Theory for Port 1 (**A**) and Port 2 (**B**). **C, D** Experiment for Port 1 (**C**) and Port 2 (**D**). For each port, the top row shows the reconstructed Stokes maps: total intensity $S_0$and the normalized parameters S_1/S_0, S_2/S_0, and S_3/S_0(color scale from −1 to +1). Overlaid markers on S_0visualize the local polarization state (ellipse/major-axis orientation). The bottom row shows the six raw analyzer images used for reconstruction: I_H, I_V, I_{+45}, I_{-45}, I_R, and I_L. Scalebar: 10 μm.

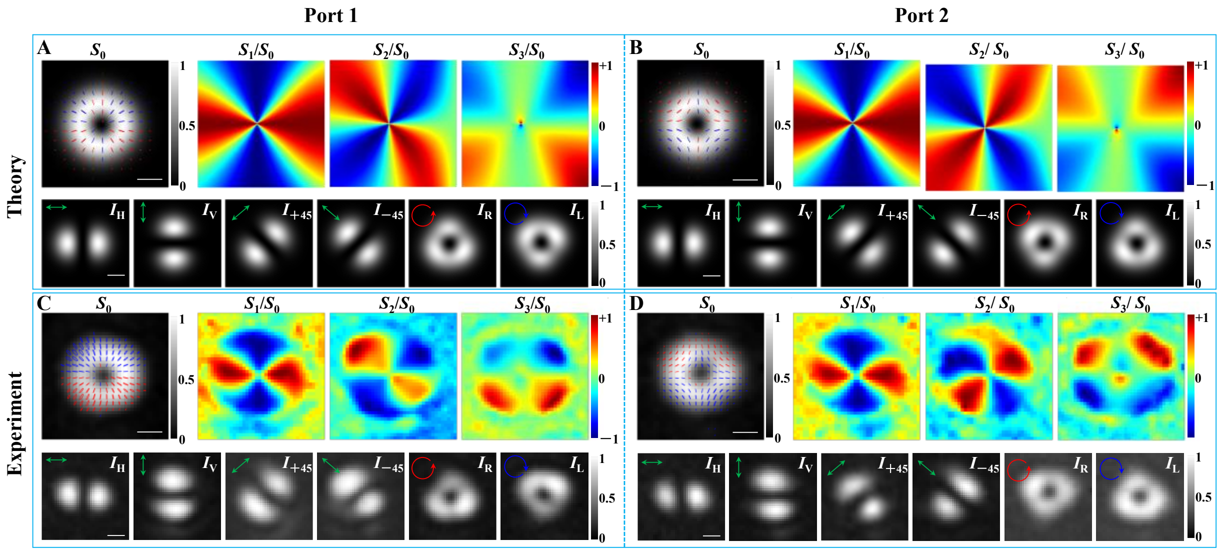

Fig. S4. Full-Stokes parameter maps across the output beam profiles at 1550 nm.

A, B Theory for Port 1 (**A**) and Port 2 (**B**). **C, D** Experiment for Port 1 (**C**) and Port 2 (**D**). For each port, the top row shows the reconstructed Stokes maps: total intensity $S_0$and the normalized parameters S_1/S_0, S_2/S_0, and S_3/S_0(color scale from −1 to +1). Overlaid markers on S_0visualize the local polarization state (ellipse/major-axis orientation). The bottom row shows the six raw analyzer images used for reconstruction: I_H, I_V, I_{+45}, I_{-45}, I_R, and I_L. Scalebar: 10 µm.

To quantify polarization crosstalk (extinction) versus $\Delta\varphi$. We choose one spin as the intended spin mode (e.g., σ^+) and measure the transmitted power $P_{\text{intended}}(\Delta\varphi)$, and set the opposite spin (e.g., σ^-) as the unintended spin mode and measure the residual transmitted power $P_{\text{unintended}}(\Delta\varphi)$. The polarization crosstalk (extinction) is defined as$10\log_{10}[P_{\text{intended}}(\Delta\varphi)/\ P_{\text{unintended}}(\Delta\varphi)]$. Therefore, directly quantifies the effective polarization crosstalk/extinction of the spin selection (i.e., how much of the orthogonal spin component leaks through after choosing a specific spin component). We repeat the measurement with the two spins swapped (σ^- intended, σ^+ unintended) to obtain the corresponding curve for the opposite spin.

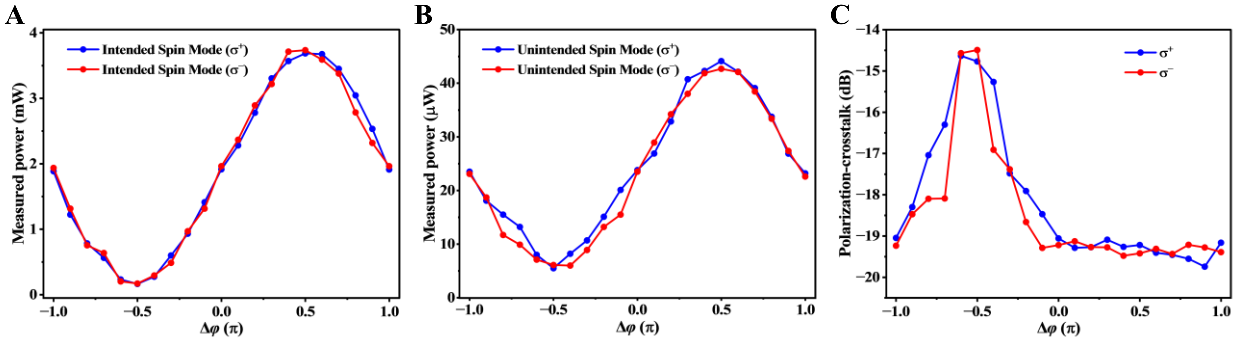

Fig. S5. Polarization-crosstalk (extinction) as a function of $\Delta\varphi$ at $\lambda = 1064$ nm

A Measured intended-spin power $P_{\text{intended}}(\Delta\varphi)$ for σ^+ (blue) and σ^- (red). **B** Measured leakage power $P_{\text{unintended}}(\Delta\varphi)$. **C** Polarization crosstalk in dB, $\text{XT}_{\text{pol}}(\Delta\varphi) = 10\log_{10}[P_{\text{unintended}}/P_{\text{intended}}]$, for both spin selections.

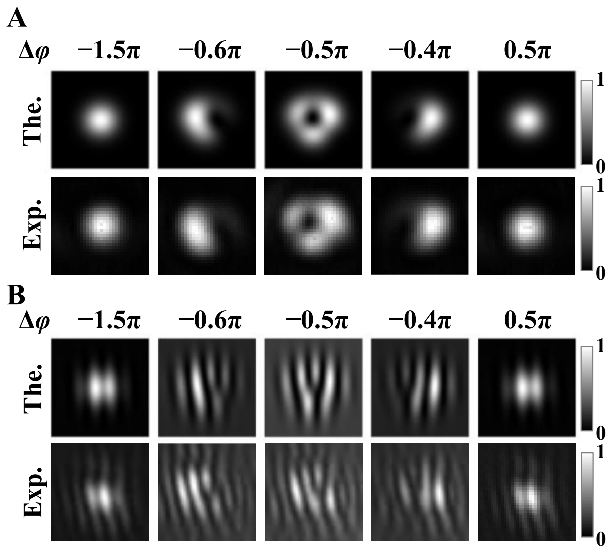

Fig. S6. The evolution of the output beam profiles for the $\sigma-$ component as the relative phase delay $\Delta\varphi$ is varied from -1.5π to $+0.5\pi$, highlighting the reversible transformation between Gaussian and vortex beams. The interferograms confirm the corresponding transitions of the topological charge from $\ell = 0$ to $\ell = -1$ and back, further verifying dynamic spin–orbit modulation for the opposite spin state.

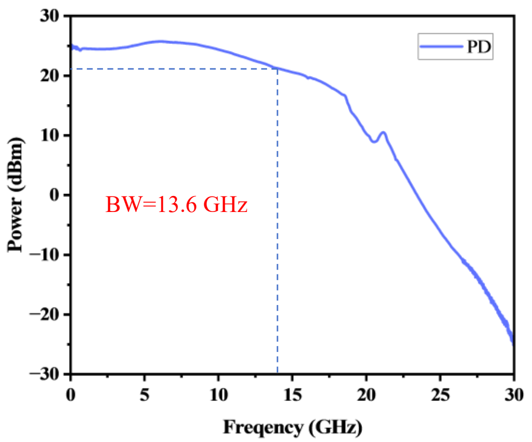

Fig. S7. Measured frequency response of the photodetector used in the dynamic measurements, which shows a 3 dB bandwidth of 13.6 GHz.

The key router metrics: insertion loss (IL), contrast ratio (CR), and port/mode crosstalk (XT) for the dual-port spin–OAM routing shown in Fig. 6, are evaluated at the two operating setpoints $\Delta\varphi = \pm\pi/2$, where the Gaussian/vortex outputs swap between Port 1 and Port 2.

We define the insertion loss as (*33*):

$$\mathrm{IL} = 10\log_{10}\left(\frac{P_{\mathrm{in}}}{P_{\mathrm{out}}}\right) = -10\log_{10}\left(\frac{P_{\mathrm{out}}}{P_{\mathrm{in}}}\right),$$

where P_{out} is the total output power summed over Port 1 and Port 2, and P_{in} is the total input power.

For mode-resolved metrics, we denote the OAM mode index by $\ell \in \{0, -1, +1\}$ and the output port index by $j \in \{1,2\}$. The contrast ratio for a given (ℓ, j) is defined as

$$\mathrm{CR}_{\ell,j} = 10\log_{10}\left(\frac{P_{\ell,j}^{\mathrm{ON}}}{P_{\ell,j}^{\mathrm{OFF}}}\right),$$

where $P_{\ell,j}^{\mathrm{ON}}$ and $P_{\ell,j}^{\mathrm{OFF}}$ are taken at the two setpoints $\Delta\varphi = \pm\pi/2$, corresponding to the intended and suppressed routing states for that (ℓ, j), respectively. The port/mode crosstalk at a fixed setpoint is defined as

$$\mathrm{XT}_{\ell}(\Delta\phi) = 10\log_{10}\left(\frac{P_{\ell,\text{unintended}}(\Delta\phi)}{P_{\ell,\text{intended}}(\Delta\phi)}\right),$$

where "intended/unintended" refer to the two output ports for the same OAM mode ℓ under a fixed $\Delta\varphi$. (Therefore, more negative XT indicates better port isolation.)

Experimentally, IL is obtained directly from the total power summed over the two ports and normalized to the input. CR and XT are measured in a mode-resolved manner using vortex phase plate (VPP) based mode projection followed by single-mode-fiber (SMF) coupling, where the coupled power yields $P_{\ell,j}$ for each mode and port. At $\lambda = 1064$nm, we obtain IL ≈ 0.28 dB at both setpoints, and the corresponding mode-resolved CR and XT values are summarized in Table S1 and Table S2, respectively. The full $\Delta\varphi$-dependent power exchange and insertion-loss behavior are provided in Fig. S7.

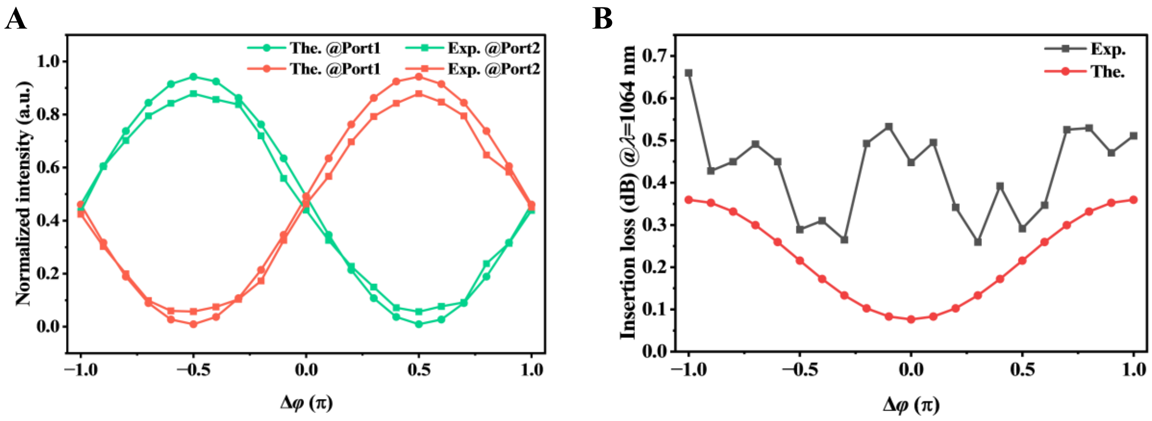

Fig. S8. Phase-dependent routing characteristics and insertion loss of the dual-port spin–OAM router (λ = 1064 nm). A Output power at Port 1 and Port 2 (normalized by the input power) as a function of the relative phase delay $\Delta\varphi$ (axis normalized to π), showing complementary sinusoidal energy exchange between the two ports. **B** Insertion loss IL($\Delta\varphi$) extracted from the total output power summed over both ports relative to the total input power. The operating setpoints $\Delta\varphi = \pm\pi/2$ correspond to the two routing states used in Fig. 6, where Gaussian/vortex outputs swap between Port 1 and Port 2.

Table S1. Contrast ratio of the dual-port router at the operating setpoints (λ = 1064 nm).

Port	Mode	CR (dB)
1	ℓ=0	18.06
	ℓ=−1	12.08
	ℓ=+1	13.88
2	ℓ=0	17.90
	ℓ=−1	13.76
	ℓ=+1	13.37

Table S2. Crosstalk of the dual-port router at the operating setpoints (λ = 1064 nm).

$\Delta\varphi$	Mode	XT (dB)
$+0.5\pi$	ℓ=0	-17.29
	ℓ=−1	-12.82
	ℓ=+1	-13.72
-0.5π	ℓ=0	-17.10
	ℓ=−1	-12.41
	ℓ=+1	-13.33

Movie S1. Cylindrical-vector verification at 1064 nm (Port 2).
Real-time recording of the Port-2 output beam profile while a linear polarization analyzer placed after Port 2 is rotated from $0°$to $360°$. The transmitted intensity distribution rotates with the analyzer angle, evidencing an azimuthally varying local polarization direction (cylindrical-vector character) rather than a uniformly polarized scalar beam.

Movie S2. Cylindrical-vector verification at 1550 nm (Port 2).
Real-time recording of the Port-2 output beam profile while a linear polarization analyzer placed after Port 2 is rotated from $0°$to $360°$. The transmitted intensity distribution rotates with the analyzer angle, evidencing an azimuthally varying local polarization direction (cylindrical-vector character) rather than a uniformly polarized scalar beam.

Movie S3. Long-term stability of phase-controlled spin-orbit OAM generation.
Real-time recording of the Port-2 σ^+ beam profile with the relative phase fixed at the routing setpoint $\Delta\varphi = -\pi/2$. The spatial beam pattern remains stable for 32 minutes under ambient laboratory conditions, demonstrating robust long-timescale stability without active phase-locking.
